# The combined antimicrobial activity of citrus honey and fosfomycin on multidrug resistant *Pseudomonas aeruginosa* isolates

**DOI:** 10.3934/microbiol.2020011

**Published:** 2020-06-19

**Authors:** Amira Saied M Abdelhady, Nebal Medhat Darwish, Safaa M. Abdel-Rahman, Nagwa M Abo El Magd

**Affiliations:** Department of Medical Microbiology and Immunology, Faculty of Medicine, Ain Shams University, Cairo, Egypt

**Keywords:** *Pseudomonas aeruginosa*, Honey, fosfomycin, MIC, *exoU* genotype

## Abstract

Infections with *Pseudomonas aeruginosa* (*P. aeruginosa*) have become a real fear in hospital-acquired infections, especially in critically ill and immunocompromised patients. Thus, advance of novel anti-infectives is currently pursued. The aim of the present study was to evaluate the antibacterial effect of each of citrus honey and fosfomycin in comparison to the combined effect of both of them on multidrug resistant (MDR) *P. aeruginosa*. 50 MDR *P. aeruginosa* isolates were tested for the antibacterial effect of citrus honey. Screening for potential synergistic activity of fosfomycin and honey combinations by E test. Molecular detection of the virulent exoenzyme U (*exoU*) genotype by conventional PCR was done. The present study found that 50 % (v/v) concentration of citrus honey was sufficient to inhibit the growth of most isolates (33/50, 66%). Minimal inhibitory concentration (MIC) for fosfomycin tested by E test was found to be >128 µg/mL in 50(100%) of MDR *P. aeruginosa* isolates but after repeating E test with Mueller-Hinton agar (MHA) containing sublethal concentration of citrus honey (29/50,58%) isolates were sensitive. Also, there was a significant correlation between the presence of *exoU* gene and positive synergy of citrus honey-fosfomycin combination. This study showed that citrus honey has antibacterial effect and synergy with fosfomycin antibiotic against MDR *P. aeruginosa* isolates. Also, *exoU* positive genotype is associated with MDR phenotype. In conclusion, our results revealed that the citrus honey-fosfomycin combination showed highly statistically significant effect on MDR *P*. *aeruginosa* fosfomycin susceptibility pattern. *exoU* positive *P. aeruginosa* isolates were detected mostly in burn unit and ICUs. Also, there was a statistically significant correlation between the presence of *exoU* gene and positive result of honey-fosfomycin combination E test.

## 1. Introduction

MDR *P. aeruginosa* is recognized by the Infectious Diseases Society of America (IDSA) as one of the top six pathogens threatening healthcare systems and as the identified causative agent of a broad range of hospital and community-acquired infections [1]. As *P. aeruginosa* causes extremely severe infections in immunocompromised patients and patients with compromised anatomical natural barriers as burns and cystic fibrosis, treatment of such infections can be challenging especially with the inherited antibiotic resistance among such organism [2].

Type III secretion is one of the most significant virulence factors of *P. aeruginosa* as it allows the delivery of various exotoxins as exoenzyme S, exoenzyme U (*ExoU*), exoenzyme Y, and exoenzyme T into host cells, which can facilitate the pathogen cellular invasion. Numerous studies suggest that *ExoU*-producing strains are associated with poor outcomes, resistance to many antibiotics and high mortality rates [3],[4].

For several decades, natural antimicrobial agents have been investigated to substitute current pharmaceutical antibiotics to overcome the increasing problem of multidrug resistance among bacteria [5].

The antibacterial properties of honey are due to the high osmotic nature, the naturally low pH (3.2–4.5) and the ability to produce hydrogen peroxide. The characteristic phytochemical substances in the honey as tetracycline derivatives, peroxide, fatty acids, amylase, phenols, ascorbic acid, and benzoic acid attribute to the potent bactericidal and bacteriostatic activity of honey against pathogenic bacteria [6].

As the expanding antibacterial resistance restrains the use of novel antimicrobials agents, re-introduction of older antibiotic agents as alternative option increases. Fosfomycin is an old previous and rather decommissioned antibiotic that was formerly used orally for treatment of uncomplicated urinary tract infections (UTIs) but recently it is re-introduced as potent antimicrobial against highly resistant pathogens causing difficult-to-treat-infections [7].

The persistence of antibiotics' misuse introduces a powerful selection power in the favor of emerging antibiotic resistant mutants, experiments on honey antibacterial effect indicated that most bacterial pathogens still showing high percentage of sensitivity to honey and postulated that combinations of antibiotic and honey would delay the emergence of MDR bacteria than antibiotics alone[8].

The aim of the present study was to evaluate the synergistic antibacterial effect of citrus honey and fosfomycin on MDR *P. aeruginosa*.

## 2. Materials and methods

The present study was conducted during the period from March 2018 till March 2019 on *P. aeruginosa* isolates obtained from laboratories of Ain Shams University hospitals. The study was approved by the Research Ethics Committee of Faculty of Medicine Ain Shams University (No. FMASU M D 41/2018).

### 2.1. Identification of multidrug bacterial isolates

Antibiotic susceptibility testing was done for 83 *P. aeruginosa* isolates by disk diffusion method to identify the 50 MDR *P. aeruginosa* enrolled in this study. The used antibiotics disks were piperacillin-tazobactam (100 µg), ceftazidime (30 µg), cefepime (30 µg), aztreonam (30 µg), imipenem (10 µg), meropenem (10 µg), gentamicin (10 µg), tobramycin (10 µg), amikacin (30 µg), ciprofloxacin (5µg) and levofloxacin (5µg) (Oxoid, UK). Interpretation of results was done according to CLSI (2018) [9]. *P. aeruginosa* ATCC (27853) was used as a control strain and each isolate was considered MDR if it was not susceptible to at least one agent in at least three antibiotic classes [10].

### 2.2. Detection of *exoU* gene positive MDR *P. aeruginosa* isolates

Conventional polymerase chain reaction (PCR) was done to detect the frequency of MDR *P. aeruginosa* carrying virulant *exoU* gene among the tested isolates. DNA extraction was done using Qiagen DNeasy (Qiagen, USA) according to manufacturer's instructions, amplification of *exoU* gene was carried out using the Forward primer: GATTCCATCACAGGCTCG and the Reverse primer: CTAGCAATGGCACTAATCG (Invitrogen, USA). The process and size of the amplicons were confirmed by electrophoresis on 1% agarose gel at 3,308 bp [11],[12].

### 2.3. The effect of citrus honey on MDR *P. aeruginosa*

Citrus honey was obtained from the apiary of the experimental station of the Faculty of Agriculture, Cairo University and kept in cool (2–8 °C) and dry place in the laboratory for processing. The inhibitory effect of honey on bacterial isolates was detected by the MIC test by broth tube dilution method according to *Kacaniova et al.* [13]; Overnight cultures of each bacterial isolate in nutrient broth were tested in the presence of different concentrations of honey, after consecutive serial dilutions each isolate was incubated with 100%, 50%, 25%, 12.5%, 6.25%, 3.12%, 1.56%, and 0.78% concentrations of citrus honey and test results were detected by observation of turbidity (bacterial growth). For each isolate, the MIC of honey was last tube showing clearance and the sub-lethal concentration was the first tube showing turbidity [8].

### 2.4. The effect of fosfomycin on MDR *P. aeruginosa*

Fosfomycin E test (Himedia laboratories, India) was performed to detect the effect of fosfomycin on tested MDR *P. aeruginosa* isolates (the MIC of fosfomycin alone regarding each bacterial isolate). The bacterial suspension of each isolate was calibrated to 0.5 McFarland opacity and inoculated on MHA (Oxoid, UK), in accordance with the manufacturer's recommendations the E-test fosfomycin strip (concentration range of 0.064–1024 µg/mL) was positioned. MIC values were determined after overnight incubation at 37 °C. Taking the breakpoints of the European committee on antimicrobial susceptibility testing [14] as reference, *P. aeruginosa isolates* with fosfomycin MIC ≤ 128 µg/mL were categorized as susceptible to fosfomycin; those with MICs > 128 µg/mL were categorized as resistant.

### 2.5. The effect of citrus honey-fosfomycin combination on MDR *P. aeruginosa*

To detect the combined effect of citrus honey with fosfomycin, fosfomycin E test was repeated after incorporation of the sub-lethal concentrations of citrus honey of each bacterial isolate in the MHA plate before application of fosfomycin strip, and the reading of the E test results (MIC determination) was repeated for each isolate.

### 2.6. Statistical analysis

The collected data was revised, coded, tabulated and introduced to a personal computer (PC) using statistical package for social science (SPSS 20). Data was presented and suitable analysis was done according to the type of data obtained for each parameter. Descriptive data was expressed as mean, standard deviation (±SD) and range for numerical data and as frequency and percentage of non-numerical data. Analytical statistics was done by Fisher's exact test for the relationship between tested qualitative variables and McNemar test for the statistical significance of the difference between tested qualitative variables measured for the same study group.

## 3. Results

Over 12 months, 50 MDR *P. aeruginosa* isolates were detected from 83 *P. aeruginosa* isolated from different clinical samples. Most of isolates were from pus samples (wound infection) (29/83, 35%) followed by sputum and Endotracheal Tube aspirate (16/83, 19%) each. Most of MDR *P. aeruginosa* isolates were from pus samples (wound infection) (20/50, 40%) (Table 1).

### 3.1. Antibiotic sensitivity testing

On testing for antimicrobial susceptibility, *P. aeruginosa* isolates had marked resistance to gentamicin (79%), tobramycin (78%) and fourth generation cephalosporine-cefepime (71%) (Table 2), the identified 50 MDR isolates represented 60% of the total 83 tested *P. aeruginosa isolates.* Most of MDR *P. aeruginosa* isolates were resistant to 6 classes of antibiotics (27/50, 54%), 14 MDR isolates (28%) were resistant to 5 classes of antibiotics, 6 (12%) were resistant to 3 classes of antibiotics and 3 (6%) were resistant to 4 classes of antibiotics.

### 3.2. MIC determination

The antibacterial effect (MIC) of citrus honey against MDR *P. aeruginosa* isolates revealed that 50 % (v/v) concentration of citrus honey was sufficient to inhibit the growth of most isolates (33/50, 66%) (Table 3).

**Table 1.** Distribution of *P. aeruginosa among* clinical samples.

Sample	Frequency of isolated *P. aeruginosa*	Frequency of isolated *P. aeruginosa* in health care settings	Frequency of MDR *P. aeruginosa*	Frequency of MDR *P. aeruginosa* in health care settings
Pus (wound)	29 (35%)	ICUs 2(2.4%)	20(40%)	ICUs 1(2%)
		Burn 18(21.7%)		Burn 6(12%)
		Other departments* 9(10.9%)		Other departments* 13(26%)
Endotracheal Tube (ETT)	16 (19.3%)	ICUs 16(19.3%)	9(18%)	ICUs 9(18%)
Sputum	16 (19.3%)	Outpatient departments 8 (9.6%)	10(20%)	Outpatient departments 10(20%)
		Other departments* 8 (9.6%)		
Bronchoalveolar lavage	8 (9.6%)	ICUs 8 (9.6%)	7(14%)	ICUs 7(14%)
Ear discharge	5 (6%)	Outpatient departments 5 (6 %)	0(0%)	0(0%)
Pus (joint)	4 (4.8%)	Other departments* 4 (4.8%)	3(6%)	Other departments* 3(6%)
Blood	3 (3.6%)	Other departments* 3 (3.6%)	1(2%)	Other departments* 1(2%)
Gastric biopsy	2 (2.4%)	Other departments* 2 (2.4%)	0(0%)	0(0%)
Total	83(100%)	83(100%)	50(100%)	50(100%)

*Other departments: Departments of Internal Medicine and surgery.

**Table 2.** Antibiotic susceptibility pattern of *P. aeruginosa* isolates.

Antibiotic	S	I	R
	N (%)	N (%)	N (%)
Piperacillin–tazobactam	36 (43%)	4 (5%)	43(52%)
Cefepime	23 (28%)	1 (1%)	59 (71%)
Ceftazidime	29 (35%)	2 (2%)	52 (63%)
Aztreonam	46 (55%)	9 (11%)	28 (34%)
Imipenem	44 (53%)	7 (8%)	32 (39%)
Meropenem	38 (46%)	2 (2%)	43 (52%)
Gentamicin	14 (17%)	3 (4%)	66 (79%)
Tobramycin	13 (16%)	5 (6%)	65 (78%)
Amikacin	14 (17%)	13 (16%)	56 (67%)
Ciprofloxacin	25 (30%)	5 (6%)	53 (64%)
Levofloxacin	25 (30%)	3 (4%)	55 (66%)

**Table 3.** MIC test results of citrus honey against MDR *P. aeruginosa* isolates.

Honey dilutions	1	½	1/4	1/8	1/16	1/32	1/64	1/128
MIC% (v/v)	100%	50%	25%	12.5%	6.25%	3.12%	1.56%	0.78%
No and % of *P*. *aeruginosa* isolates (Total = 50 (100%)	0 (0%)	33 (66%)	5 (10%)	9 (18%)	0(0%)	0(0%)	3 (6%)	0(0%)

### 3.3. Testing for synergistic antibiotic and honey combinations by E test

**Figure 1.**
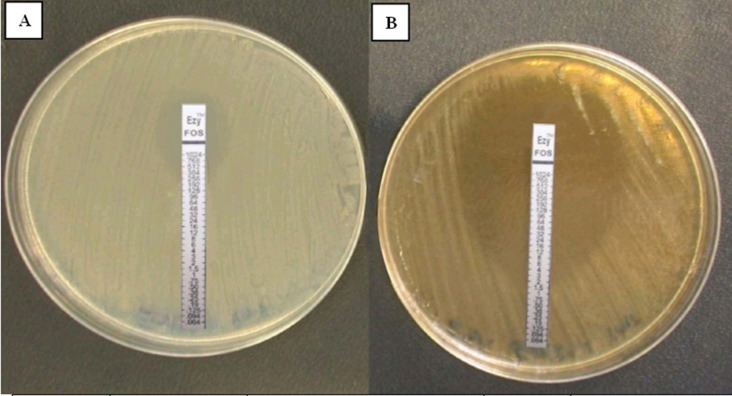
E test results of fosfomycin on honey against MDR *P. aeruginosa* isolates. A: MDR tested *P. aeruginosa* isolate to fosfomycin only on MHA (resistant to fosfomycin, MIC = 192 µg/mL). B: E test results of the same isolate on 25% honey incorporated MHA medium (sensitive to fosfomycin, MIC = 1.5 µg/mL).

All tested MDR *P. aeruginosa* isolates were resistant to fosfomycin (50/50,100%) giving MIC > 128 µg/mL by E test. On repeating fosfomycin E test with sub-inhibitory honey concentrations for each isolate incorporated in MHA (combined honey-fosfomycin effect testing), 29/50 tested isolates (58%) turned sensitive (MIC ≤ 128 µg/mL) and (21/50, 42%) only were resistant to the citrus honey-fosfomycin combination with highly statistically significant difference between E test results of fosfomycin only and combined honey-fosfomycin E test (P value < 0.001) (Table 4, Figure 1).

**Table 4.** Comparison between E test results of fosfomycin only and of combined honey-fosfomycin on MDR *P. aeruginosa* isolates.

No.of isolates	E Test fosfomycin	Combined honey-fosfomycin E test	McNemar's test	
	N 50 (100%)	N 50 (100%)	p value	sig.
Sensitive	0 (0%)	29 (58%) (Positive result)	<0.001	Highly Significant
Resistant	50 (100%)	21 (42%) (Negative result)		

### 3.4. Molecular detection of the virulent *exoU* genotype

**Figure 2.**
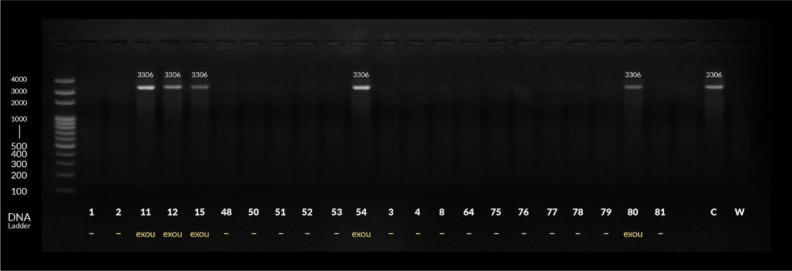
Detection of *exoU* gene by conventional PCR among MDR *P. aeruginosa.*

As regards detection of the virulent *exoU* gene among MDR *P. aeruginosa* isolates, among the tested 50 isolates, only 5 isolates were positive for *exoU* gene by PCR (10%). MDR *P. aeruginosa* isolates were mostly isolated from patients admitted to intensive care units (ICUs) (17/50, 34%), and the *exoU* gene was more frequently detected among MDR *P. aeruginosa* isolated from those patients (4/5, 80%) and also from patients admitted to the burn unit (1/5, 20%). There was a statistically significant correlation between the presence of *exoU* gene and positive result of honey-fosfomycin combination E test. All *exoU* gene positive isolates (5/5 100%) were resistant to 6 classes of the tested antibiotics but the correlation was nonsignificant (Tables 5, 6, 7, Figure 2).

**Table 5.** Distribution of *exoU* gene + ve *P. aeruginosa isolates* among health care settings.

Health care settings	*ExoU* gene – ve 45(100%)	*ExoU* gene + ve 5(100%)
Intensive care units (ICUs)	13(28.9%)	4(80%)
Burn	5(11.1%)	1(20%)
Outpatient departments	10(22.2%)	0(0%)
Other departments*	17(37.8%)	0(0%)

*Other departments: Departments of Internal Medicine and surgery.

**Table 6.** The correlation between detection of *exoU* gene and combined honey-fosfomycin E test.

		PCR for *exoU* gene		Fisher exact test	
		Negative	Positive		
		No 45(100%)	No 5(100%)	p value	sig.
Combined honey-Fosfomycin	Positive result	24 (53.3%)	5 (100%)	0.01	Significant
E test	Negative result	21 (46.7%)	0 (0%)		

**Table 7.** The correlation between detection of *exoU* gene and number of resistant classes of tested antibiotics.

		PCR for *exoU* gene		Fisher exact test	
		Negative	Positive		
		N 45(100%)	N 5(100%)	p value	sig.
No. of Resistant classes of antibiotics	3	6(13.3%)	0 (0%)	0.297	Nonsignificant
	4	3(6.7)	0 (0%)		
	5	14(31.1%)	0 (0%)		
	6	22(48.9%)	5 (100%)		

## 4. Discussion

*P. aeruginosa* is an opportunistic pathogen that can cause outbreaks of hospital-acquired and life-threatening infections, especially among immunocompromised and critically ill patients [15].

The present study was conducted on 83 isolates of *P. aeruginosa.* Most isolates were from pus samples of wound infections (29/83, 35%) followed by sputum (16/83.19%) and the least was from blood (3/83, 3.6%) and gastric biopsy (2/83, 2.4%). These results agree with Hashem et al. [16] as they stated that most of isolated *P. aeruginosa* isolates were from wound specimens (63/147 isolates, 43%) and sputum 23% (34/147 isolates), Al-Haik et al. [17] also found in his study that the highest isolation rate was (60%) from wounds and burns patients followed by sputum (26.7%), and the lowest rate from blood(16.6%), the highest incidence of *P. aeruginosa* (47.5%) was stated in ICU in agree with our results as 26 of the isolated 83 *P. aeruginosa* (31.3%) were from patients admitted to the ICU. Bashir et al. [18] also found that the highest incidence of *P. aeruginosa* (47.5%) was found in ICU, which was followed by endoscopy unit and female surgical wards (40%). Intensive care patients are more susceptible to infection because of the debilitating effect of a prolonged hospitalization and instrumentation [17].

Increasing rates of MDR *P. aeruginosa* in healthcare associated infections (HAIs) and among hospitalized patients is a chief public health problem [19]. Resistance of *P. aeruginosa* is usually accompanied by the production of biofilms, active expulsion of antibiotics by efflux pump, and alteration of outer membrane protein expression [20].

The present study demonstrated that *P. aeruginosa* isolates had marked resistance to gentamicin (79%), tobramycin (78%) and fourth generation cephalosporine, cefepime (71%). Less resistance was noticed for aztreonam (55 %) and carbapenem antibiotics imipenem and meropenem were 53% and 46% respectively. Similar results were reported by Kamel et al. [21] as isolates were totally resistant to tobramycin and gentamicin and were sensitive to amikacin (68%), imipenem and meropenem(52%) and ciprofloxacin (36%), and by Mansour et al. [22] who found that resistance to aztreonam was the highest (96.6%), followed by cefepime (76.3%) and tobramycin (67.8%). Ayatollahi et al. [23] study in Iran found that *P. aeruginosa* isolates were most sensitive to imipenem (55%) and then to amikacin (45%). Moreover, the highest resistance was to ceftriaxone (100%). An Indian study by Dash et al. [24] showed that the majority of *P. aeruginosa* isolates were resistant to ceftazidime 70%, followed by cefepime 64.8%, piperacillin 45%, ciprofloxacin 38.9%, levofloxacin 36.1%, gentamicin 37.3% and amikacin 30%. The differences in antibiotic susceptibility pattern in different regions could be attributed to the differences in the study population, the duration of hospitalization, cross-infection, the dose and types of antibiotics commonly used, in addition to the difference in adherence level to antimicrobial policies controlling the spread of MDR organisms [17].

The present study also found that (50/83, 60%) of the tested *P. aeruginosa* isolates were MDR, this agrees with many studies performed in Egypt by Farhan et al. [25]; Hashem et al. [16] and Raouf et al. [26] who found MDR strains in 66.6%, 64% and 97% of the *P. aeruginosa* isolates respectively. But our results disagree with those of Sader et al. [27] and Samad et al. [28] in United States of America (USA) and Pakistan who reported that MDR strains were 15.4% and 39.44% among *P. aeruginosa* isolates, respectively. The increasing level of resistance in MDR *P. aeruginosa* is often attributed to patient-to-patient transmission of resistant strains as well as newly acquired resistance owing to previous antibiotic exposure [19].

The quantity and presence of established antimicrobial factors in honey varies widely, which may influence overall effectiveness. Diverse Studies have found that honeys from specific floral sources present stronger antibacterial activity than other types of honey [29],[30].

According to Hegazi [31] and Roby et al. [32], the citrus honey has strong antibacterial action compared to different types of tested honey. The present study showed that 50 % (v/v) concentration of citrus honey was sufficient to inhibit the growth of most isolates (33/50, 66%) that is similar to studies done by Wasihun and Kasa [6], Jantakee and Tragoolpua [33] who reported that the percentage by volume of honeys to prevent growth of *P. aeruginosa* ranged from 12.5% to 50% v/v for most tested bacterial isolates. In contrast, Ahmed et al. [34] found that honey from Gondar Zuria district in Ethiopia showed bacteriostatic effect against *P. aeruginosa* at 6.25% v/v. Another study by Mandal and Mandal [35] found that The MICs of different honeys against clinical and environmental isolates of *P. aeruginosa* was recorded as 12.5% v/v. This variability in results could be clarified by the fact that different honeys vary in their antibacterial potency, which may be owing to variations in plant source or the geographical distribution. Most of literatures that discussed the use of honey in treating microbial infection did not explain enough the type of honey used. Other factors that may affect honey activity are seasonal changes, harvesting, processing, and storage conditions of the tested honeys. Consequently, honeys are not equal in their antimicrobial effectiveness [36],[37].

This difference in the antibacterial activity of honeys over place may also be because of the difference in the species of bees and the differences in the test methods used and test organisms, where in our study we used MDR bacteria [6].

In latest decades there has been a remarkable increase in resistance to currently available antibiotics and a marked decline in discovery and development of novel antibiotics, clinicians have been forced to reconsider the therapeutic potential of older, underutilized antibiotics such as fosfomycin for the treatment of infections caused by MDR Gram-negative organisms especially as part of combination therapy [1]. The present study discovered that MIC for fosfomycin tested by E test was found to be >128 µg/mL in all tested MDR *P. aeruginosa* isolates (50/50, 100%). These results agree with the Egyptian study done by Behera et al. [38] on non-urinary MDR isolates of *P. aeruginosa* who reported non susceptibility of (17/25, 68%) to fosfomycin and also with Perdigão-Neto et al. [39] in Brazil who reported that 14 of 15 (93%) MDR isolates was fosfomycin non susceptible, whereas a study by Walsh et al. [1] in United Kingdom reported that fosfomycin susceptibility was 21 of 37 (57%) MDR *P. aeruginosa* isolates and also Maraki et al. [40] in Greece reported 8 of 9 (89%) MDR strains were susceptible which is much higher susceptibility percent than our study. Variable results clearly highlight the need for more uniformity of testing methods (disc diffusion, agar dilution, broth microdilution and E test) as well as information on the pharmacodynamics of fosfomycin for *P. aeruginosa* so that organizations such as EUCAST will be able to create effective breakpoint criteria. They also reinforce the need to determine local susceptibility patterns [1].

It has been revealed that combinations of antibiotics with non-antibiotic substances can improve the efficacy of a number of currently used antibiotics by forming synergetic combinations [41],[42]. Synergistic combinations exhibit a decrease in the MIC value of the antibiotic when combined with honey. This would allow for dose reduction of the antibiotic thereby minimising possible side effects, reducing treatment costs and providing a therapeutic option with greater antimicrobial potential. Furthermore, the potential risk of antimicrobial resistance is thought to be minimised with the utilisation of combination therapy [43].

Fosfomycin was initially isolated from cultures of Streptomyces species in 1969. Its reported mechanism of action is to disrupt the formation of the peptidoglycan precursor uridine diphosphate N-acetylmuramic acid, the first cytoplasmic step in the biosynthesis of the bacterial cell wall [1]. This single mechanism of action denotes that cross-resistance with other classes of antibiotics is less likely and allows fosfomycin to retain significant in vitro activity against numerous Gram-positive and Gram-negative bacteria, including MDR strains. Based on this action, interest in fosfomycin has increased among clinicians and microbiologists worldwide for all potential facets of use [44].

Fosfomycin activity against nonfermenting Gram-negative bacteria such as *P. aeruginosa* and *Acinetobacter baumannii* in conditions of multidrug resistance is less predictable and differs widely depending on the phenotypes present in the various epidemiological environments [45],[46].This antibiotic's specific mechanism of action makes it a highly attractive option for use in combination with other agents based on the synergy or addition observed in *in vitro* studies [44].

According to the present study 50(100%) MDR *P. aeruginosa* isolates were resistant to fosfomycin when tested by E test alone but after repeating E test with MHA containing sublethal concentration of citrus honey, (29/50,58%) isolates were sensitive and (21/50, 42%) only were resistant to the citrus honey-fosfomycin combination with highly statistically significant difference (P value ≤ 0.05), Khan et al. [43] showed in their study that synergism between South African honeys and the broad-spectrum antibiotic ciprofloxacin against *P. aeruginosa* was statistically highly significant, another study by Jenkins and Cooper [8] showed that tetracycline and colistin showed improved activity in the presence of honey against *P. aeruginosa.*

The *exoU* gene is responsible for a highly cytotoxic phenotype which leads to host cell death and is considered to be a significant factor implicated in the severity of infections caused by *P. aeruginosa* and as an independent factor of early mortality during blood infections [47].

The present study found that only 5/50 (10%) of the MDR *P. aeruginosa* isolates harbored the *exoU* gene.

This result is in agreement with Bradbury et al. [48] and Hassuna [3] who found that the frequency of the *exoU* gene was (18%), however, it was relatively lower than that reported by Horna et al. [4] and Mitov et al. [49] with a frequency of 22.7% and 30% respectively.

The present study revealed that the *exoU* gene was more frequently detected among MDR *P. aeruginosa* isolated from patients attended ICUs (4/5, 80%) and the burn ward (1/5, 20%).These results in conformity with Horna et al. [4] who found that *exoU* gene was more frequent among *P. aeruginosa* from patients attending ICUs (9/18, 50.0%) and the burn ward (6/8, 75.0%). These data suggest that *exoU* positive genotype might be genetically favored in environments with high antibiotic pressure, such as ICUs so the *exoU* positive isolates were more prone to be associated with ICU and burn wards, and subsequently with the most fragile patients of hospital environment [48].

In the present study, a significant correlation have been found between the presence of *exoU* gene and positive citrus honey-fosfomycin combination test results, such findings encourage the therapeutic usage of citrus honey-fosfomycin combination in critical cases in ICUs and burn units where higher percent of *exoU* gene positive isolates exist as more positive results will be attained in favor of patients' improved morbidities and in decreasing rates of MDR *P. aeruginosa* strains.

All *exoU* gene positive isolates were resistant to more than 3 classes of the tested antibiotics but the correlation was nonsignificant. This result in agreement with Horna et al. [4] who found that Overall, 33 out of 43 *exoU* positive isolates were classified as MDR so the presence of *exoU* was significantly associated with MDR (20.9%). Also, our results agree to Mitov et al. [49] who found that the frequency of the *exoU* gene were significantly higher in MDR strains than those in non-MDR *P. aeruginosa* strains.

This association between the *exoU* genotype and the MDR phenotypes could be because of the presence of transferable antibiotic-resistant determinants such as integrons carrying mobile gene cassettes within the accessory genome of *exoU* positive *P*. *aeruginosa* [50].

## 5. Conclusion

The citrus honey-fosfomycin combination showed highly statistically significant effect on MDR *P*. *aeruginosa* fosfomycin susceptibility pattern. *exoU* positive *P. aeruginosa* isolates were detected mostly in burn unit and ICUs. Also, there was a statistically significant correlation between the presence of *exoU* gene and positive result of honey-fosfomycin combination E test. Larger scale studies are recommended to explain the exact concentration citrus honey-fosfomycin combination that provide the desired therapeutic effects on different infections caused by MDR *P*. *aeruginosa.*
